# Hypertensive Emergency As Initial Presentation of Systemic Sclerosis Sine Scleroderma

**DOI:** 10.7759/cureus.37817

**Published:** 2023-04-19

**Authors:** Sumugdha Rayamajhi, Shailendra Sharma, Subash Regmi, Farah M Anwar, Hardik Gandhi

**Affiliations:** 1 Internal Medicine, Michigan State University College of Human Medicine, East Lansing, USA; 2 Nephrology, Sparrow Health System, Lansing, USA; 3 Nephrology, University of Southern Carolina, Columbia, USA; 4 Internal Medicine, Michigan State University, East Lansing, USA

**Keywords:** severe hypertension, sine scleroderma, systemic sclerosis, scleroderma, hypertensive emergency

## Abstract

Hypertensive emergency is a common cause of emergency room (ER) visits. Scleroderma renal crisis (SRC) is one of the rare causes of hypertensive emergency. SRC is a life-threatening condition that presents with acute onset severe hypertension accompanied by retinopathy, encephalopathy, and rapidly worsening renal function. We present a case of hypertensive emergency and renal failure with positive anti-Scl 70 and RNA polymerase III which is characteristic of SRC. Despite appropriate supportive care and timely treatment with angiotensin-converting enzyme inhibitors, the patient progressed to end-stage kidney disease.

## Introduction

Hypertensive emergency accounts for significant visits to the emergency room with high acuity levels. It is important for clinicians to be aware of unusual causes of hypertensive emergencies. Scleroderma renal crisis (SRC) is a complication of systemic sclerosis (SS) that causes accelerated phase hypertension (HTN) and acute renal failure [1]. While SRC presents years following the onset of SS, it can also present in the absence of cutaneous and digital findings of SS, a condition termed systemic sclerosis sine scleroderma (ssSSc) [1]. Here we present a case of SRC presenting as a hypertensive emergency in a patient without a prior diagnosis of SS.

## Case presentation

A 53-year-old African American female with a history of diabetes mellitus, HTN, morbid obesity, and chronic kidney disease (CKD) stage 3 was seen in the emergency department for progressive shortness of breath. Initial evaluation showed blood pressure (BP) of 253/122, heart rate (HR) of 81/min, respiratory rate (RR) of 24/min, and oxygen saturation of 80%. She was awake but in significant respiratory distress with bilateral extremity edema and bibasilar crackles. She did not have a rash, joint pains, photosensitivity, oral ulcers, hair loss, dysphagia, Raynaud’s phenomenon, or skin tightening.

She was admitted to ICU for a hypertensive emergency with acute hypoxic and hypercarbic respiratory failure, acute on chronic renal failure, and acute pulmonary edema. She was placed on bilevel positive airway pressure (BiPAP) to help with the work of breathing and the nitroglycerin drip started. Nitroglycerin drip was later switched to clevidipine drip and home medications were resumed with some adjustments, which included carvedilol 25 mg, clonidine 0.2 mg, isosorbide mononitrate 30 mg, hydralazine 100 mg, nifedipine 90 mg, and furosemide 40 mg. Clonidine 0.2 mg patch. Based on the information provided by the patient’s family, the patient was consistently noncompliant with medications.

She presented evidence of end-organ dysfunction including acute on chronic kidney injury and acute pulmonary edema. Pertinent labs are shown in Table 1.

**Table 1:** Lab values BNP: brain natriuretic peptide; pCO2: partial pressure of carbon dioxide; Hgb: hemoglobin; ANA: anti-nuclear antibody; IgG: immunoglobulin G; MPO: myeloperoxidase; anti-CCP: anti-cyclic citrullinated peptide; anti-SSA: anti-Sjogren's syndrome A; anti-SSB: anti-Sjogren's syndrome B; anti-Sm: anti-Smith; anti-RNP: antinuclear ribonucleoprotein

Lab findings	Results	Normal range	Units
Serum Creatinine	3.32	1.3-1.5	mg/dL
BNP	429	<100	pg/mL
pH	7.19	7.35-7.45	N/A
pCO2	53	35-45	N/A
Hgb	6.9	14-17	g/dL
Platelets	177 x 10(3)	150-450 x 10(3)	uL
ANA	Positive (1:640) Homogenous	< 1:80	neither speckled nor nucleated immunofluorescence pattern
Anti-Scl-70s	Positive	<29	AU/mL
RNA polymerase III Ab IgG	Weakly Positive (31.6)	<20	U
IgG	1780	600-1500	mg/dL
MPO	1.9	0-469	pmol/L
C3	Normal	0.88-2.01	g/dL
C4	Normal	0.15-0.45	g/dL
IgA	Normal	61-356	mg/dL
IgM	Normal	37-286	mg/dL
Anti-CCP	Negative	<20	Unit
Anti-SSA SSB	Negative	<7	U/mL
Anti-Sm/RNP	Negative	0-7	U/mL
Anti-Jo1	Negative	<29	Unit

There was no evidence of microangiopathic hemolytic anemia with normal lactate dehydrogenase, normal bilirubin, and absence of schistocytes on the peripheral blood smear. The hepatitis panel was negative. Urinalysis showed small blood, 41-50 WBC per high power field, and urine protein to creatinine ratio of 9.5 (<0.2). Given acute on chronic renal insufficiency and difficult-to-control HTN, an autoimmune workup was pursued which showed positive antinuclear antibody (ANA) and negative Anti dsDNA. The rest of the serologic workup was performed as shown in Table 1. Renal ultrasound was performed which showed non-echogenic normal-sized kidneys and a transthoracic echocardiogram was unremarkable with mild left ventricular hypertrophy (LVH) and preserved ejection fraction.

A diagnosis of SRC was made based on positive serologic markers anti-Scl 70 and RNA polymerase III along with worsening renal failure and severe uncontrolled HTN. In the absence of cutaneous findings and digital vasculopathy, it was further categorized as having SRC sine scleroderma. She was started on captopril at 6.25 mg TID which was titrated to 12.5 mg TID. BP management continued to be a challenge despite the addition of vasodilators including minoxidil, hydralazine, isosorbide mononitrate, and diuretics. Kidney function failed to improve despite aggressive supportive measures and she eventually developed end-stage kidney failure and was started on hemodialysis.

## Discussion

SRC occurs most frequently with diffuse scleroderma and is a relatively early complication of SSc, commonly presenting within the first four years of clinical diagnosis. The triggers for SRC remain unclear, but several risk factors for SRC have been identified, including diffuse cutaneous disease, the presence of tendon friction rub, arthritis, and the positivity of anti-RNA-polymerase III antibody [1]. Glucocorticoid use is strongly associated with SRC [1-3] with approximately 60% of patients with SRC having had prior and recent exposure to glucocorticoids. We describe a case of ssSSc presenting as SRC.

Pathology

The injury in SRC is the culmination of vasospasm superimposed on the uncontrolled accumulation of collagen and intimal proliferation of the arteries, with concentric onion skin hypertrophy. This results in the narrowing or obliteration of the vascular lumen. Resultant ischemia of the juxtaglomerular apparatus (JGA) causes enhanced renin secretion and formation of angiotensin II further worsening vasoconstriction. The disease process is localized in small arcuate and interlobular arteries and glomeruli. Autopsy studies suggest 60-80% of patients with diffuse cutaneous SSc have pathologic evidence of kidney damage [4] and that SRC develops in approximately 10-15% of patients with scleroderma [1,4-6]. However, visceral organ involvement may occur in the absence of cutaneous manifestations, in the condition referred to as SSc sine scleroderma. Kidney biopsy is generally not required for enabling diagnosis but may be helpful for predicting clinical outcomes and reversibility of renal failure [7].

Hypertensive emergency

Hypertensive emergencies are defined as marked BP elevations (typically >180 mm Hg systolic or >110 mm Hg diastolic) associated with acute target organ damage in the cardiovascular, renal, and central nervous systems [8]. Adult ED visits have more than doubled in recent years [9]. HTN emergencies overwhelm our healthcare systems and can make up as much as one-fourth of all ED visits, with as much as 0.3% of all emergency room visits [8,9].

Scleroderma renal crisis (SRC)

SRC presents with acute onset severe HTN accompanied by retinopathy, encephalopathy, and rapidly worsening renal function. The presentation is usually years following the onset of SSc. However, SRC can occur in the absence of cutaneous and digital findings of SSc as well. The prevalence of SRC can be between 2% and 15% of all patients with SSc [10]. It is a potentially fatal complication if left untreated and is associated with a very poor renal prognosis. Without treatment, SRC can progress to end-stage renal disease (ESRD) over a period of 1-2 months and death usually occurs within one year [4]. Once a fatal disease, the outcome of patients with SRC has changed with the advent of angiotensin-converting enzyme inhibitor (ACE-i) therapy and dialysis. However, success with antihypertensive therapy is dependent upon early initiation before irreversible renal damage has occurred. Treatment with ACE-i has been associated with greater BP control, preservation of renal function, and better patient survival with SRC [1,11,12].

Systemic Sclerosis Sine Scleroderma (ssSSc)

ssSSc is a rare subset of SSc characterized by the total or partial absence of cutaneous manifestations of SSc with the occurrence of internal organ involvement and serologic abnormalities [13]. Diagnosis of ssSSC requires a very high index of suspicion. Our case illustrates challenges associated with diagnosing SRC, especially in the complete absence of cutaneous involvement and digital vasculopathy as has been reported previously.

Early recognition and intervention

Diagnosis of SRC can be missed more so in the absence of cutaneous changes. The incidence of SRC decreased by over 50% between 2002 and 2013, while the prevalence increased by about 10% [14]. This signals some success in both renal replacement therapy (RRT) and rapid treatment of SSc-associated malignant HTN with ACE-i. Success with antihypertensive therapy is dependent upon early initiation before irreversible renal damage has occurred. ACE-i are associated with greater BP control, preservation of renal function, and improved survival in patients with SRC [1,11,12]. Captopril has a rapid onset and short duration of action and should be the mainstay of treatment for all patients, regardless if they are normotensive or hypertensive, with the goal of bringing BP back to baseline within 72 hours. Monitoring of serum creatinine in patients on ACEi is crucial because SRC is essentially a form of bilateral intrarenal artery stenosis.

Although ACE-i is the mainstay of treatment, there is conflicting data on the preventative role of ACE-i. A large retrospective study found a decreased risk of SRC among SSC patients treated with dihydropyridine calcium channel blocker (CCB) [13].

SRC outcome

SRC is a potentially fatal complication if left untreated. It is associated with a very poor renal prognosis. The prevalence of this condition can be between 2% and 15% of all patients with SSc [10]. Without treatment, SRC can progress to ESRD over a period of 1-2 months with death usually occurring within one year [4]. Once a fatal disease, the outcome of patients with SRC has changed with the advent of ACE-i therapy and dialysis. Success with antihypertensive therapy is dependent upon early initiation before irreversible renal damage has occurred. Treatment with ACE-i has been associated with greater BP control, preservation of renal function, and better patient survival with SRC [1,11,12].

Scleroderma renal crisis and renal replacement therapies

Despite treatment with ACE-i, approximately 20% of patients with SRC end up requiring RRT [1,11]. Hruskova et al. have identified that the perceived possibility of recovery with SRC has resulted in a long time on dialysis before being offered transplantation, 256 days compared to 112-163 days (P<0.001) for control groups. Survival on dialysis in patients with SRC is worse than in other forms of ESRD [15,16]. Patient survival after a kidney transplant is superior to that in dialysis patients with SRC who remain on the waiting list [16,17]. Maintenance immunosuppression regimens include low-dose corticosteroids, mycophenolate, and sirolimus (with the goal of avoiding higher-dose corticosteroids and calcineurin inhibitors). SRC recurs in less than 5% of patients who receive a kidney transplant. Early loss of native kidney function due to SRC appears to be a risk factor for the recurrence of SRC in transplanted kidneys.

## Conclusions

We present an unusual case of hypertensive emergency due to SRC without evidence of scleroderma. The low prevalence of SRC could be attributed to underreporting in the literature. Our goal is to contribute to limited literature and increase awareness among clinicians to consider this diagnosis in patients presenting with hypertensive emergencies with initial negative workup. A high index of suspicion is warranted among clinicians for early recognition and intervention which may prevent mortality and morbidity in this population.
